# Elevated levels of Merkel cell polyoma virus in the anophthalmic conjunctiva

**DOI:** 10.1038/s41598-021-92642-w

**Published:** 2021-07-28

**Authors:** Nora Siegal, Michal Gutowski, Lakshmi Akileswaran, Norman J. Beauchamp, Lien-Chieh Ding, Christopher B. Chambers, Russell N. Van Gelder

**Affiliations:** 1 Department of Ophthalmology, University of Washington School of Medicine, Campus Box 359608, 325 9th Avenue, Seattle, WA 98104 USA; 2 Department of Ophthalmology, Oregon Health Sciences University, Portland, OR USA; 3 Department of Biological Structure, University of Washington School of Medicine, Seattle, WA USA; 4 Department of Laboratory Medicine and Pathology, University of Washington School of Medicine, Seattle, WA USA

**Keywords:** Infection, Conjunctival diseases

## Abstract

The human ocular surface hosts a paucibacterial resident microbiome and virome. The factors contributing to homeostasis of this mucosal community are presently unknown. To determine the impact of ocular enucleation and prosthesis placement on the ocular surface microbiome, we sampled conjunctival swabs from 20 anophthalmic and 20 fellow-eye intact conjunctiva. DNA was extracted and subjected to quantitative 16S rDNA PCR, biome representational karyotyping (BRiSK), and quantitative PCR (qPCR) confirmation of specific organisms. 16S ribosomal qPCR revealed equivalent bacterial loads between conditions. Biome representational in silico karyotyping (BRiSK) demonstrated comparable bacterial fauna between anophthalmic and intact conjunctiva. Both torque teno virus and Merkel cell polyoma virus (MCPyV) were detected frequently in healthy and anophthalmic conjunctiva. By qPCR, MCPyV was detected in 19/20 anophthalmic samples compared with 5/20 fellow eyes. MCPyV copy number averaged 891 copies/ng in anophthalmic conjunctiva compared with 193 copies/ng in fellow eyes (*p* < 0.001). These results suggest that enucleation and prosthesis placement affect the ocular surface flora, particularly for the resident virome. As MCPyV has been shown to be the etiologic cause of Merkel cell carcinoma, understanding the mechanisms by which the ocular surface regulates this virus may have clinical importance.

## Introduction

Human mucosal surfaces are normally colonized by a diverse community of microorganisms. In recent years, the mucosal microbiome has been extensively studied in tissues including the oral mucosa, vaginal mucosa, and nasopharynx^1^. The ocular surface is mucosal, but unlike other mucosa, has a relatively paucibacterial resident microbiome consisting primarily of low numbers of coagulase-negative *Staphylococcus, Cutibacteria* (formerly *Propionibacteria),* and *Corynebacteria*^2–7^. The bacterial load of the conjunctiva is approximately 1% that of oral mucosa or skin^2,3^. Multiple mechanisms have been hypothesized to account for the paucity of bacteria on the ocular surface, including the mechanical action of eyelid blinking, the presence of lysozyme and lactoferrin in tears, and the abundance of IgA in tears^8,9^. Recent research has also demonstrated a resident conjunctival virome including torque teno virus (TTV), a small single stranded DNA anellovirus^3,10,11^ and Merkel cell polyoma virus (MCPyV), a small double stranded DNA polyomavirus^3^. Both TTV and MCPyV have also recently been described in intraocular fluids from post-operative endophthalmitis cases^10,12^.

Previous studies have suggested that the ocular microbiome is disrupted and re-organized following enucleation^13,14^. The anophthalmic socket may be altered by absence of normal ocular tissues such as the cornea; by presence of a prosthesis; by medication history preceding and following enucleation; and by the underlying condition necessitating enucleation or evisceration. Prior studies have used standard bacterial culture techniques to characterize changes in ocular surface flora of the anophthalmic socket and found increased microbial density and a modest shift toward more pathogenic organisms. The purpose of the present study was to apply molecular DNA detection techniques, which have the advantages of being able to detect unculturable or poorly culturable bacteria as well as fungi, parasites and DNA viruses, to compare the anophthalmic conjunctiva with intact fellow eyes.

## Methods

### Study subjects

Institutional approval for this study was obtained from the University of Washington Institutional Review Board. This study followed the tenets of Helsinki as well as ARVO guidelines for research. This study was a registered clinical trial at ClinicalTrials.gov (NCT02306668). Written informed consent was obtained from all subjects.

20 subjects with unilateral anophthalmia were recruited from the oculofacial plastic and reconstructive surgery service of the University of Washington Medicine Eye Institute. Inclusion criteria were history of enucleation or anophthalmia with prosthesis and scleral shell placement in one eye, and ability to provide consent. Exclusion criterion was evidence of active infection at time of sampling; and any evidence of acute prosthetic malfunction such as erosion or extrusion. All subjects wore ocular prostheses (scleral shell) at the time of this study, which was removed aseptically immediately before sampling. After instillation of sterile, topical proparacaine, DNA swabs (Isohelix, SK-2S) were used to obtain samples from the lower conjunctival fornices of the anophthalmic and fellow-eye conjunctiva for molecular analysis. Genomic DNA was isolated from the conjunctiva swabs using the DNeasy Blood & Tissue Kit using manufacturer’s suggested protocol (Qiagen, Inc., Venlo, Netherlands). DNA was quantified using the Qubit® dsDNA HS Assay Kit (ThermoFisher Scientific, Waltham, MA).

### Molecular analysis

To calculate bacterial load in each sample, pan-bacterial PCR was performed using 16S ribosomal DNA universal primers as well as primers for human actin to normalize for total DNA recovery (Integrated DNA Technologies, San Diego, CA) as described previously^3^. Quantitative PCR was used to confirm presence of TTV as described previously^11^. Primer sequences are given in Table 1. All quantitative PCR assays were performed on the Applied Biosystems 7500 Fast Real-Time PCR system (Foster City, CA) for 30 cycles and quantified using standard curves of cloned positive control template. The final PCR mix contained 0.8 µL each of forward and reverse primers (final concentration of each, 0.4 mM), 10  µL of the FastStart SYBR Green Master Mix without ROX (Roche, Basel, Switzerland), and 2 µL of unamplified genomic DNA. Master mix was prepared under a laminar hood to minimize contaminations. The final reaction volume was 20 µL. For standard curve, a plasmid complementary DNA of the cloned region of interest (e.g., the target sequence for 16S, MCPyV, TTV, or actin) was serially diluted tenfold to obtain copy numbers ranging from 10^1^ to 10^8^ copy/mL. Quantitative PCR routinely was able to detect 10 copies/mL of each control complementary DNA. The PCR reaction consisted of initial holding stage at 50 °C for 2 min and at 95 °C for 10 min, followed by a cycling stage at 95 °C for 15 s and 60 °C for 1 min. 30 cycles of amplification were performed. Copy number of experimental samples was calculated by interpolation of delta CT number against the standard curve derived from the cloned product. Double-distilled H_2_O was used as negative control in all experiments, and was tested at time of PCR reaction. Water/air controls were not obtained at the time of swabbing.

**Table 1:** PCR primer sequences.

Gene/organism	Forward primer	Reverse primer
β-actin	5′-TGCTCCTCCTGAGCGCAAG-3′	5′-GCCGGACTCGTCATACTCC-3′
16S	5′-GAGGAAGGTGGGGGATGACGT-3′	5′-AGGCCCCGGGAACGTATTCAC-3′
TTV	5′-AGGTGAGTTTACACACCGCAGTCA-3′	5′-AATGAAGACCCTAAGAGCCTTGCC-3′
MCPyV	5′-TTGTCTCGCCAGCATTGTAG-3′	5′-GGTGCAGATGCAGTAAGCAG -3′

Biome representational in silico karyotyping (BRiSK) is a whole genome representational deep sequencing technique that allows for an unbiased characterization of the DNA-based metagenomic constituents in biopsy samples^15^. In brief, a type IIS restriction endonuclease was used to isolate 33 bp fragments associated with a specific 6-mer recognition sequence in the DNA and subjected to deep DNA sequencing. Genomic DNA was subjected to whole genome amplification using phi29 polymerase and digested with BsaXI. Illumina-compatible barcoded paired-end adapters were ligated to digested DNA, followed by isolation of sequences with asymmetric adapters using streptavidin column and PCR-amplified using adapter sequences. The resulting product was gel-purified and subjected to high throughput sequencing on the Illumina MiSeq sequencer (Illumina, San Diego, CA). BRiSK data were analyzed for presence of bacteria, fungi and viruses as described previously^11^. DNA tags were cross-referenced to a comprehensive database for taxonomic classification.

### Statistical analysis

Statistical analyses were performed using SPSS for Windows software version 26 (IBM Corporation, Armonk, North Castle, NY, USA). Descriptive analyses are reported as means and standard deviation for continuous variables and as percentage for qualitative variables. All continuous and paired statistical analyses were completed by the related-samples Wilcoxon signed rank test, while the categorical data were analyzed by Fisher’s Exact Test. A 2-tailed probability of 0.05 or less was considered statistically significant.

## Results

Demographic data for subjects in this study are shown in Table 2. Among the 20 participants (7 female, 13 male) the average age was 59.5 ± 15 years (mean ± standard deviation). The most common cause of anophthalmia was trauma (40%) but also included previous corneal damage (20%), birth defects (microphthalmos 20%) and retinoblastoma (5%). Mean time since anophthalmic surgery was 26.1 ± 18 years with current prosthetic mean age of 6.7 ± 9 years. The majority of patients (70%) did not endorse symptoms in their anophthalmic socket (Table 3). For those who did, discharge and erythema of the conjunctiva were most common (both 10%). 75% of patients were not using any ocular treatments in their anophthalmic socket. Topical medication use for the remainder included erythromycin (10%), artificial tears (5%), and blephamide (5%). One subject was taking systemic methotrexate (5%). 75% of patients did not have a significant ocular history in their contralateral eye. No subject had evidence of active infection at the time of sampling.

**Table 2:** Patient characteristics.

**Total sample size**	20
**Age, mean (SD)**	60 (15)
**Gender, n (%)**	
Female	7 (35%)
Male	13 (65%)
**Race, n (%)**	
American African	2 (10%)
Asian	1 (5%)
Latino	1 (5%)
White	16 (80%)
**Reason for prosthesis, n (%)**	
Anophthalmos	1 (5%)
Blind/painful	1 (5%)
Corneal scar	4 (20%)
Glaucoma	1 (5%)
Melanoma	1 (5%)
Microphthalmos	2 (10%)
Retinoblastoma	1 (5%)
Trauma	8 (40%)
Tumor	1 (5%)
**Surgery, n (%)**	
Enucleation	12 (60%)
Evisceration	1 (5%)
Unknown	7 (35%)
**Years after surgery, mean (SD)**	26 (18)
**Eye, n (%)**	
OD	8 (40%)
OS	12 (60%)
**Prostheses age (years), mean (SD)**	7 (9)

**Table 3:** Clinical history.

**Recent symptoms, n (%)**	
Discharge	2 (10%)
Erythema	2 (10%)
Tearing	1 (5%)
None	14 (70%)
**Medication use, n (%)**	
Artificial tears	1 (5%)
Blephamide	1 (5%)
Erythromycin	2 (10%)
Methotrexate PO	1 (5%)
None	15 (75%)
**Contralateral eye history, n (%)**	
Cornea transplant	1 (5%)
Dry eye	1 (5%)
Glaucoma	1 (5%)
Microphthalmos	1 (5%)
Uveitis	1 (5%)
None	15 (75%)

**Figure 1:**
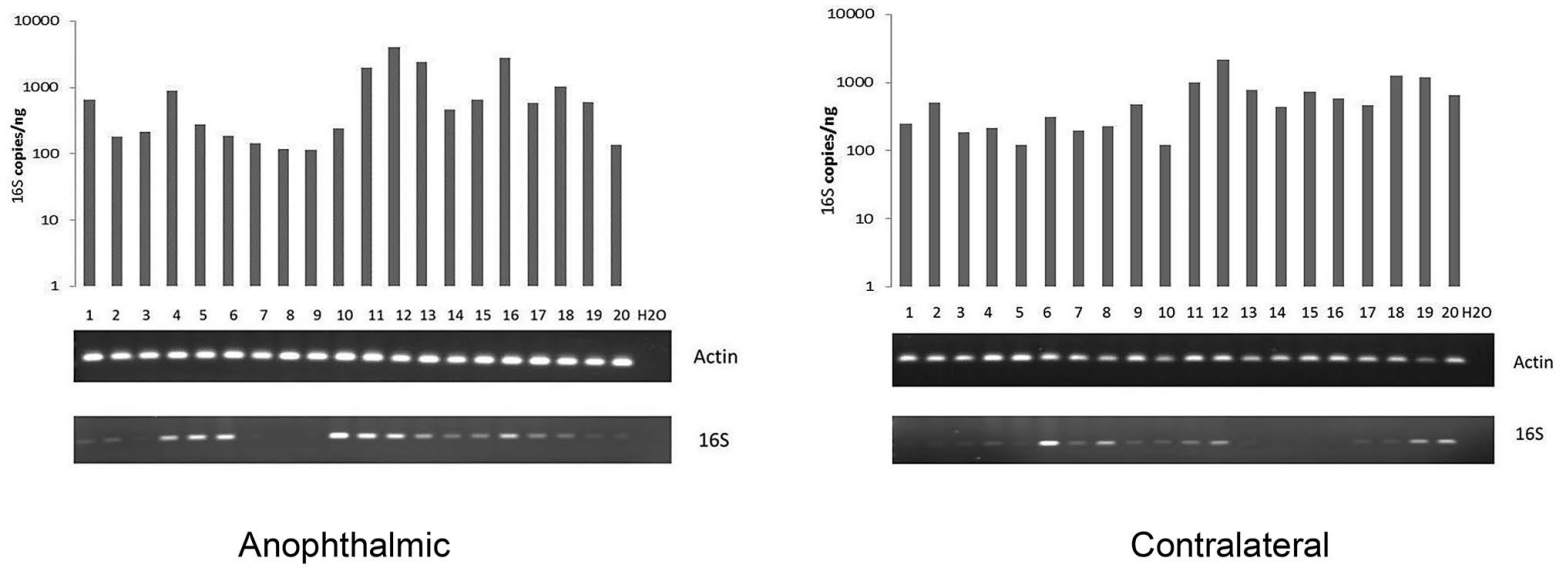
Quantitative (top) and qualitative PCR for 16S ribosomal DNA from anophthalmic (left) and contralateral (right) conjunctiva. Lower images are from ethidium-bromide stained gel electrophoresis. H20 = double distilled water negative control.

We first sought to determine the overall bacterial load present on anophthalmic and intact conjunctiva using quantitative 16S universal bacterial PCR. Actin was used as positive control for DNA extraction. As shown in Fig. 1, all samples showed robust amplification for actin indicating sufficient sample acquisition. Double distilled water was used as negative control in all experiments and yielded no amplification product. Quantitative PCR for actin revealed no difference in recovery between anophthalmic and control conjunctiva (Table 3). Qualitative 16S PCR demonstrated presence of bacteria in 16/20 anophthalmic samples and 15/20 contralateral samples (Fig. 1). Quantitative PCR was performed for 16S rDNA. By this more sensitive technique, all samples showed products, generally ranging between 100 and 1000 copies/ng starting DNA. Normalization of 16S quantitative PCR for actin revealed average bacterial loads of ~ 0.2 16S copies per actin copy (Table 4). As bacteria have an average of ~ 4 16S copies per cell^16^, this suggests an average bacterial load of ~ 1 bacterium per 10 human cells in both groups (on the same order of magnitude as reported in previous studies of healthy conjunctiva^3^). No significant difference in normalized bacterial load was observed in anophthalmic compared with control conjunctiva (0.229 vs. 0.225, *p* = 0.765 by related-samples Wilcoxon signed rank test).

**Table 4:** Quantitative PCR in anophthalmic and contralateral conjunctiva.

**Copies of Actin per Nanogram of Conjunctival Sample DNA**				
Anophthalmic Conj. actin/ng	Control actin/ng	Paired Diff		
Mean (SD)	Mean (SD)	Mean (SD)	*p* value	95% CI
4490.364 (3657.661)	3653.227 (1453.032)	837.138 (3260.157)	0.526	(-688.662,2362.939)
**Anophthalmic 16S/actin vs. Control 16S/actin**				
Anophthalmic Conj	Healthy Conj	Paired Diff		
Mean (SD)	Mean (SD)	Mean (SD)	*p* value	95% CI
0.229 (0.226)	0.225 (0.381)	0.004 (0.279)	0.765	(-0.127,0.134)
**Copies of MCPyV per nanogram of conjunctival sample DNA**				
Anophthalmic Conj MCPyV/ng	Healthy Conj MCPyV/ng	Paired Diff		
Mean (SD)	Mean (SD)	Mean (SD)	*p* value	95% CI
891.770 (349.725)	193.053 (376.000)	698.717 (343.792)	** < 0.001**	(537.817,859.617)
**Copies of MCPyV normalized to actin: MCPyV/Actin**				
Anophthalmic Conj	Healthy Conj	Paired Diff		
Mean (SD)	Mean (SD)	Mean (SD)	*p* value	95%CI
0.427 (0.423)	0.060 (0.120)	0.366 (0.415)	** < 0.001**	(0.172,0.520)
**Copies of TTV per nanogram of conjunctival sample DNA**				
Anophthalmic Conj	Healthy Conj	Paired Diff		
Mean (SD)	Mean (SD)	Mean (SD)	*p* value	95% CI
41.148 (151.640)	8.042 (12.549)	33.105 (140.235)	0.232	(-32.527,98.738)

16S metagenomic sequencing is prone to substantial artifact when applied to samples with low bacterial loads, including amplification of environmental DNA and erroneously high estimates of diversity^17^. BRiSK is a representational deep DNA sequencing technique in which ~ 1% of all DNA in a sample is sequenced, and the presence of non-human DNA is catalogued^15^. This technique is capable of detecting any DNA-based life-form, including bacteria, fungi, parasites, and DNA viruses, and has been used to characterize the healthy human microbiome at the genus level^3,18^. Because of potential biases in amplification, it is not a quantitative technique when applied to samples with low bacterial loads, but may be considered similar to culture, which is generally reported as present vs. absent. Presence of bacteria using BRiSK in conjunctival samples from the anophthalmic socket vs. the healthy contralateral socket is shown in Table 5. In both the healthy and anophthalmic sockets, the most common bacterial genera detected were *Corynebacterium* (9/20 anophthalmic vs 8/20 contralateral), *Staphylococcus* (8/20 anophthalmic vs 10/20 contralateral), and *Cutibacterium* (formerly *Propionibacteria*, 8/20 anophthalmic vs 4/20 anophthalmic). None of these differences were statistically significant by Fisher exact test. Of note, two anophthalmic sockets had detectable *Clostridium* sequences while none was seen in contralateral control eyes. *Clostridium* sequences have been detected occasionally in previous studies as a component of normal conjunctiva^3,19^.

**Table 5:** BRiSK Detection of Bacteria and Viruses in Anophthalmic vs. Contralateral Socket.

Organisms	Anophthalmic socket		Contralateral Healthy eye	
	N	%	N	%
**Bacteria**				
*Corynebacteria*	9	45	8	40
*Cutibacteria*	8	40	4	20
*Staphylococci*	8	40	10	50
*Clostridia*	4	20		
*Streptococci*	4	20	2	10
*Streptomyces*	2	10	2	10
*Rothia*			2	10
**Viruses**				
Torque teno virus	7	35	4	20
Merkel cell polyomavirus	5	25	2	10
Proprionibacterium phage	5	25	1	5
Betapapilloma virus	4	20	3	15
SEN virus	4	20	1	5
Human endogenous retrovirus H	3	15	1	5
Human papilloma virus	3	15	1	5
Gammapapilloma virus	2	10		
Human herpes virus	1	5		
Human polyoma virus	1	5		

**Figure 2:**
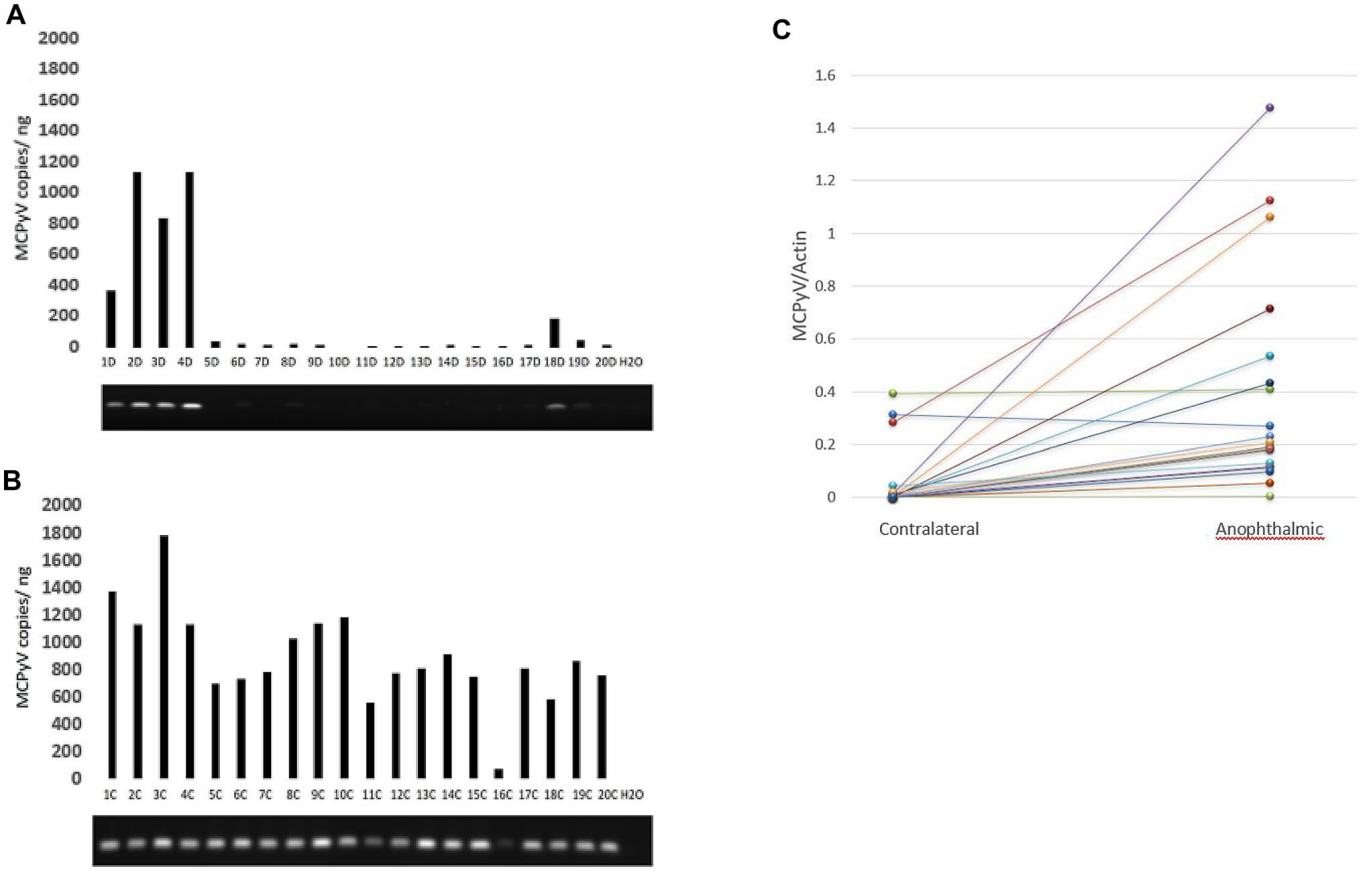
A. Quantitative and qualitative PCR for Merkel cell polyoma virus (MCPyV) in control conjunctiva. B. Quantitative and qualitative PCR for MCPyV in conjunctiva of anopththalmic eyes of same subjects. H_2_0 = double distilled water negative control. C. Comparison of quantitative MCPyV viral load normalized to actin (see Fig. 1) from anophthalmic and contralateral eyes.

Four DNA viruses – TTV, MCPyV, SEN virus (a small DNA virus closely related to TTV^20^), and papilloma-family viruses – were found by BRiSK in at least some samples (Table 5). All were found more commonly in the anophthalmic socket than in the contralateral socket. More sensitive quantitative PCR was used to determine the number of copies of MCPyV and of TTV from the anophthalmic socket compared to the healthy contralateral conjunctiva (Table 3 and Fig. 2). 19 of 20 samples demonstrated higher quantitative load of MCPyV in the anophthalmic conjunctiva than in the contralateral conjunctiva (*p* < 0.0001 by Fisher exact test). Quantitative PCR demonstrated an average of 891 MCPyV copies/ng DNA in the anophthalmic conjunctiva compared to 193 MCPyV copies/ng DNA on the healthy contralateral conjunctiva (Fig. 2, *p* < 0.001 by related-samples Wilcoxon signed rank test). TTV copy number was higher in the anophthalmic conjunctiva than the contralateral conjunctiva in a majority of cases (13/20), and TTV copies/ng were higher in anophthalmic vs. contralateral conjunctiva (41 copies/ng vs 8 copies/ng), but this result was not statistically significant on paired significance testing (*p* = 0.232).

## Discussion

Characterizing the normal microbiome of the ocular surface, specifically the bulbar and palpebral conjunctivae, is important for understanding the etiology of ocular infections (such as corneal ulcers and post-operative endophthalmitis) and ocular inflammatory conditions (such as keratitis)^4^. While traditional conjunctival culture techniques have consistently identified several common ocular resident bacterial species, 16S rDNA PCR deep sequencing has revealed the presence of additional bacteria and, surprisingly, resident viruses^5,7^. The sensitivity of BRiSK to detect viruses was demonstrated by Muthappan et al.^15^ who used paraffin-embedded microscope slides of nasopharyngeal carcinoma to detect Epstein Barr Virus (HHV-4), the causative agent of this malignancy. In a more recent study, the conjunctival microbiome of healthy individuals was characterized using BRiSK analysis, with results consistent with the hypothesis that there is both a bacterial and a viral community in normal human conjunctiva^3^. The bacterial community is described as paucibacterial, with less than one bacterial genome recovered per 10 human cells. This is approximately two orders of magnitude lower bacterial load than is seen in buccal mucosa or skin. A subsequent study using qPCR of 16S DNA suggested even lower bacterial load in the eyes of contact lens wearers, on the order of one bacterium per 500 human cells^18^. The most prevalent bacterial genera identified in conjunctiva in these studies were *Corynebacterium*, *Cutibacterium (Propionibacterium)*, *Staphylococcus* and *Streptococcus*. TTV was also identified by BRiSK on the ocular surface^3,18^, and was confirmed to be present by PCR in approximately half of ocular surface samples. Doan et al. also detected MCPyV and papilloma viruses on healthy conjunctiva in small subsets of patients^3^.

The goal of the present study was application of this type of molecular microbiome analysis to the conjunctiva of the anophthalmic socket. The anophthalmic socket is categorically different from that of normal ocular adnexa. In most cases it has undergone trauma (enucleation or evisceration, and often surgeries such as ruptured globe repair which predated the removal of the globe), has been exposed to topical antibiotics, and no longer has normal corneal tissue present. In the current study, we find these changes have minimal long-term impact on the bacterial components of the conjunctival surface, with no significant changes seen either in the load of bacteria present (as measured by quantitative 16S PCR) nor their general type. These results differ somewhat with the findings of Toribio et al.^13,14^, who found greater microbial density and modestly increased prevalence of gram-negative bacteria and coagulase-positive *Staphylococcus* on the anophthalmic conjunctival surface using standard culture techniques. This discrepancy likely has several causes. The overall bacterial load found by BRiSK is largely made up of *Cutibacteria* (previously named *Propionibacteria*^21^) and *Corynebacteria* species. These species are recovered poorly in culture and so are likely underestimated, but are fully represented by BRiSK and quantitative 16S PCR. Additionally, BRiSK is unable to distinguish bacteria at the species level, and the technique cannot reliably distinguish coagulase-positive from coagulase-negative *Staphylococcus.* Our results were consistent with Toribio et al. in finding increased prevalence of *Streptococcus* in anophthalmic sockets; however, in the present study this did not reach statistical significance as it did in their larger study (which looked at over 180 anophthalmic surfaces). Overall, however, our results suggest that the conjunctival surface in the anophthalmic socket does not have grossly altered bacterial flora in either quantity or general constituency, but may have more subtle changes in the bacterial microbiome.

The current study did demonstrate significantly higher recovery of several viruses from the anophthalmic conjunctiva than the contralateral eye. MCPyV, TTV, SEN virus, and papilloma viruses were all identified by BRiSK more frequently from anophthalmic conjunctiva than from contralateral tissue. Quantitative PCR demonstrated substantially higher recovery of MCPyV DNA from the anophthalmic conjunctiva, with ~ fourfold higher average viral copy number. While higher recovery of TTV was also noted, this result was not statistically significant in our relatively small study.

MCPyV belongs to the *Polyomaviridae* family of viruses which are icosahedral double-stranded DNA viruses. MCPyV DNA is normally present in many locations in humans including the GI tract^22^, lymphoid tissues^23^, urine^24^, the respiratory tract^25^, and saliva^26^. In these locations, the virus does not appear to induce malignancy with significant frequency, suggesting that it is part of a normal virome. MCPyV has been shown to be a causative agent in Merkel cell carcinoma (MCC)^27–29^, a rare but often aggressive neuroendocrine tumor of the skin. Consistent with a viral etiology for cancer, MCC is significantly more common in immunosuppressed individuals such as those with HIV^30^. Higher loads of MCPyV have been observed in normal skin of patients with MCC than in matched controls, suggesting the hypothesis that higher viral loads predispose to disease^31^. MCC affects the eyelids in approximately 2.5% of cases^32^, and typically affects patients in their 6th decade or older^33^. This tumor has a poor prognosis with approximately 40% mortality. As the eyelids represent only ~ 0.03% of the surface area of human skin, MCC is over-represented in eyelid tumors by about 100-fold. It is conceivable that this may be related to the presence of MCPyV on the ocular surface. While our review of the literature did not yield any cases of MCC in lids of previously enucleated eyes, given the rarity of this tumor (fewer than 50 cases of eyelid MCC annually in the US^32^) and the relative rarity of enucleation and evisceration (rate of ~ 2.5/100,000 in one survey in the United Kingdom^34^ suggesting prevalence of anophthalmia of less than 1/1,000) we would calculate expectation of fewer than one case of MCC in the lid of an enucleated individual every 20 years. The fact that enucleation appears to dysregulate MCPyV on the ocular surface suggests that other factors may also affect levels of this virus, and could constitute risk factors accounting for the high relative frequency of this tumor on the eyelids.

The current study has several limitations. This study had a relatively small sample size which, as noted previously, may have limited power to measure subtle changes in the bacterial microbiome of the anophthalmic surface. Time of day and pre-collection routine (such as face washing) were not standardized. Additionally, we did not account for systemic co-morbidities in our subjects. Systemic diseases such as diabetes mellitus could conceivably have an effect on ocular surface flora (although we used fellow eyes as control to attempt to normalize for this). The use of BRiSK and confirmatory PCR was entirely DNA-based. As such, we did not look for the presence or levels of RNA viruses on the ocular surface such as hepatitis C virus. By using DNA for our assays, we additionally cannot distinguish between live and dead bacteria in the present study. Additionally, as with any amplification-based technique, the possibility exists for contamination of reagents generating false-positive results (although this risk appears lower for BRiSK than for 16S metagenomic approaches^3^). Finally, our normalization assumes that the expression of actin per human cell remains constant following enucleation; it is conceivable that scarring could change the level of actin expression which could bias these results. However, the inability to detect MCPyV by PCR in the majority of contralateral eyes suggests the observed viral load difference between fellow eyes is substantial.

In conclusion, the current study demonstrates the diverse ocular surface microbiome and virome of the anophthalmic socket. In comparison to the healthy ocular surface, MCPyV is a more frequent component of the anophthalmic virome. The factors contributing to higher recovery of MCPyV in anophthalmic sockets remain to be identified, but these results suggest the normal eye contributes to regulation of ocular surface flora, particularly its virome. The effects of these viruses on the normal and abnormal conjunctiva remains to be determined.

## Supplementary Information

Supplementary Information.
