# Supplementary material for: Elevated levels of Merkel cell polyoma virus in the anophthalmic conjunctiva
Source: Sci Rep. 2021 Jul 28;11:15366. doi: 10.1038/s41598-021-92642-w (PMC8319407; doi:10.1038/s41598-021-92642-w)

As recommended by *Nature* journals, we include the uncropped gel images, taken directly from photographics of ethidium bromide agarose gel electrophoresis. In each case, upper tier is anophthalmic socket and lower is contralateral eye.

1.) Actin PCR

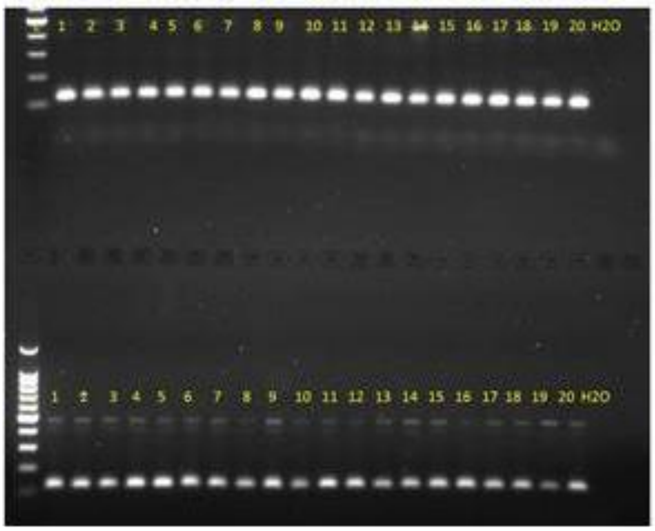

2.) 16S PCR

16S direct PCR Anophthalmic samples C &D

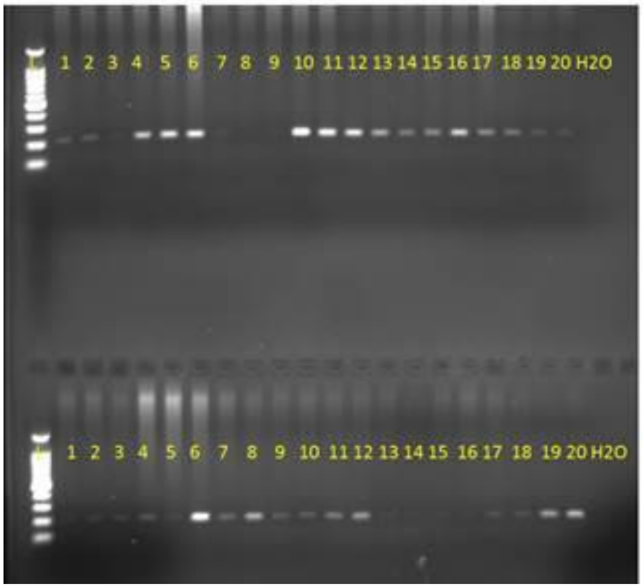

3.) Merkel cell polyoma virus

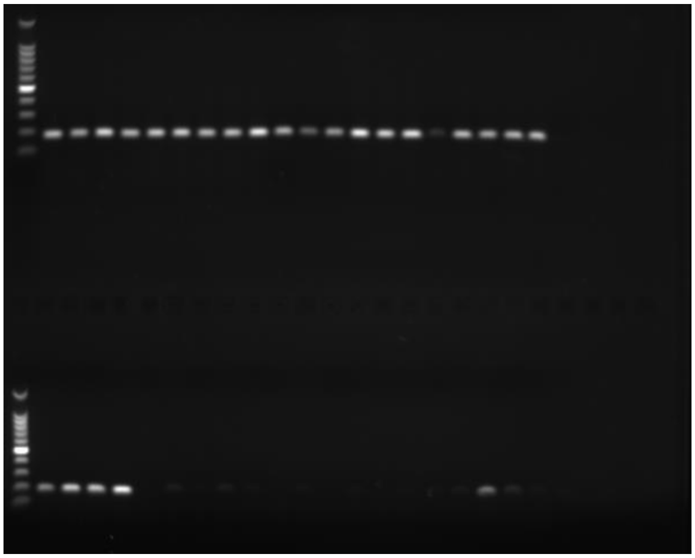

Supplement: Supplementary file 1 — Supplementary Information. [file 41598_2021_92642_MOESM1_ESM.pdf]
